# The complete plastome of bloodroot (*Sanguinaria canadensis*, Papaveraceae), a spring ephemeral from eastern North America

**DOI:** 10.1080/23802359.2019.1662747

**Published:** 2019-09-09

**Authors:** Zihan Liu, Yuxin Luo, Yuguo Wang, Joongku Lee

**Affiliations:** a Laboratory of Systematic and Evolutionary Botany and Biodiversity, College of Life Sciences, Zhejiang University, Hangzhou, Zhejiang, P. R. China;; b Ministry of Education, Key Laboratory for Biodiversity Science and Ecological Engineering, Institute of Biodiversity Science, Fudan University, Shanghai, P. R. China;; c Department of Environment and Forest Resources, Chungnam National University, Daejeon, South Korea

**Keywords:** Chelidonieae, Papaveroideae, plastome, *Sanguinaria*

## Abstract

The complete plastome of *Sanguinaria canadensis* was sequenced, which is 160,117 bp in length, consisting of 87699 bp large single-copy (LSC), a 19,136 bp small single copy (SSC), and a pair of 26,638 bp inverted repeat (IR) regions. The *S. canadensis* plastome encodes 137 annotated genes including 37 tRNA genes, 8 rRNA genes, and 89 protein coding genes. Phylogenetic analysis strongly supported that it is a member of Chelidonieae.

*Sanguinaria canadensis* belongs to Papaveroideae, a subfamily sister to Fumarioideae in the poppy family (Hoot et al. 2015). *Sanguinaria canadensis*, or bloodroot for common name, is a spring ephemeral herb typically growing in the understory of temperate deciduous forests (Blattner and Kadereit 1999). To avoid dense canopy of deciduous woody plants in warm growing seasons, spring ephemerals take advantage of the high-level sunlight in spring, during when they complete their life cycles, prior to the formation of canopy (Archibold 1995). This strategy is strongly associated with the temperate deciduous forest, which makes *S. canadensis* a good model to study on the formation and evolution of the North American temperate deciduous forest biome. There have been few researches on Papaveroideae and its species (Hoot et al. 1997; Schwarzbach and Kadereit 1999; Guo et al. 2014; Yun and Oh 2018 Geneious Prime). But till now, no plastome of *S. canadensis* is available.

Bloodroot collected from Marianna, Florida, USA (N30°48′16.9″, W85°13′36.5″, collection NO.: *Pan Li LP162495*) was selected for genome skimming analysis. The voucher specimen was stored in the Herbarium of Zhejiang University (HZU), Hangzhou, Zhejiang, China. Fresh leaves were dried with silica gel and total genomic DNA was extracted using Plant DNAzol Reagent and sequenced using the Illumina Hiseq 2500 (Illumina, San Diego, CA, USA), with 150 bp paired-end sequencing. The raw reads were deposited in the National Center for Biotechnology Information (NCBI) Sequence Read Archive (BioProject ID: PRJNA512066). GetOrganelle (Jin et al. 2018) was used to construct the complete plastome sequence. The plastome was annotated using Geneious Prime 2019.1.1 (www.geneious.com) as described in Liu et al. (2017), and finally submitted to GenBank (MN241447).

The complete plastome of *S. canadensis* is 160,117 bp in length, consisting of a 87,699 bp LSC (large single-copy) region, a 19,136 bp SSC (small single-copy) region, and a pair of 26,638 bp IR (inverted repeat) regions. In total, 134 genes were annotated including 37 tRNA genes, 8 rRNA genes, and 89 protein-coding genes. 20 of all genes (7 tRNA genes, 4 rRNA genes, and 9 protein-coding genes) duplicate in IR regions, while the others exhibit single copies. The average GC content of overall cp genome is 38.5%. For LSC, SSC, and IR regions, GC contents are 8, 32.5, and 8%, respectively.

Complete plastome sequences of *S. canadensis* and 15 other species in Ranunculales were employed to infer the systematic position of *S. canadensis* (Figure 1). Using RAxML Black Box on CIPRES science gateway (Stamatakis 2014), we generated a maximum likelihood (ML) tree. A species from Eupteleaceae (*Euptelea pleiosperma*) was chosen as outgroup according to Hoot et al. (2015). The phylogenetic analysis strongly supported that *S. canadensis* is a member of Chelidonieae.

**Figure 1.**
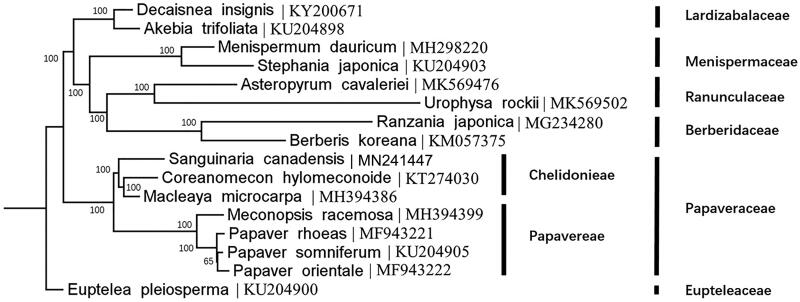
The maximum likelihood (ML) tree based on 16 complete plastome sequences of Ranunculales with 1000 bootstrap replicates.
